# Risk factors associated with diabetes after liver transplant

**DOI:** 10.20945/2359-3997000000447

**Published:** 2022-03-23

**Authors:** Mariana Baldini Campos, Cínthia Minatel Riguetto, Ilka de Fátima Santana Ferreira Boin, Arnaldo Moura

**Affiliations:** 1 Universidade de Campinas Faculdade de Ciências Médicas Departamento de Clínica Médica Campinas SP Brasil Disciplina de Endocrinologia, Departamento de Clínica Médica, Faculdade de Ciências Médicas, Universidade de Campinas, Campinas, SP, Brasil; 2 Universidade Estadual de Campinas Faculdade de Ciências Médicas Departamento de Cirurgia Campinas SP Brasil Departamento de Cirurgia, Faculdade de Ciências Médicas, Universidade Estadual de Campinas, Campinas, SP, Brasil

**Keywords:** Post-transplant diabetes mellitus, liver transplantation, immunosuppressive drugs, insulin resistance, diabetes mellitus

## Abstract

**Objective:**

Post-transplant diabetes mellitus (PTDM) is a common metabolic complication after liver transplant that negatively affects a recipient's survival and graft function. This study aims to identify risk factors associated with diabetes after liver transplant.

**Materials and methods:**

This is a cross-sectional study conducted from September to November 2019. Data collection was performed by chart review, and patients were divided into 3 groups: patients without diabetes mellitus (DM), patients with pre-transplant diabetes mellitus, and patients with PTDM.

**Results:**

Two hundred and forty-seven patients’ medical charts were screened, and 207 patients were included: 107 without DM, 42 with pre-transplant DM, and 58 with PTDM. The leading cause for liver transplant was hepatitis C, followed by hepatocellular carcinoma secondary to alcohol. There was a higher exposure to tacrolimus in patients without DM ( *P* = 0.02) and to ciclosporin in patients with pre-transplant DM, compared to others ( *P* = 0.005). Microscopic interface inflammatory activity was more severe in patients without DM as well as those with PTDM ( *P* = 0.032). There was a higher prevalence of steatosis in recipients with pre-transplant DM than there was in others ( *P* < 0.001). Multivariate logistic regression identified the following independent risk factors for DM: cirrhosis due to alcohol, hepatitis C, and triglycerides. For PTDM, these independent risk factors were cirrhosis due to alcohol, hepatitis C, and prednisone exposure.

**Conclusion:**

Alcoholic cirrhosis is a risk factor for PTDM in liver recipients. Liver transplant recipients with a pre-transplant history of cirrhosis due to alcohol, hepatitis C, and prednisone exposure deserve more caution during PTDM screening.

## INTRODUCTION

Post-transplant diabetes mellitus (PTDM) is the most common metabolic complication in the postoperative period after a liver transplant. PTDM can negatively affect a recipient's survival rate and graft function. It also has a considerable negative influence on postoperative rejection, incidence of cardiovascular diseases, infection, and neuropsychiatric problems ( 1 , 2 ). The term PTDM refers to diabetes mellitus (DM) diagnosed in the post-transplant period, regardless of the time of occurrence, covering clinically stable patients who have developed persistent hyperglycemia ( 3 ). It occurs in 10% to 40% of patients who receive solid organ transplants, depending on the transplanted organ, genetic predisposition, and patient's age ( 4 ). Orthotopic liver transplant is the treatment of choice for irreversible liver failure ( 5 ). The main indications are alcoholic cirrhosis, viral hepatitis, hepatocellular carcinoma (HCC), acute liver failure, and cirrhosis due to nonalcoholic fatty liver disease. Other less common indications that result from progressive liver failure are primary biliary cirrhosis, cryptogenic cirrhosis, autoimmune hepatitis, primary sclerosing cholangitis, hepatopulmonary syndrome, Caroli disease, Wilson's disease, hemochromatosis, and Budd Chiari syndrome ( 5 ). With the worldwide obesity pandemic, nonalcoholic fatty liver disease is becoming one of the leading causes of liver cirrhosis and transplantation.

Cardiovascular events are more frequent in liver transplant recipients who have post-transplant metabolic syndrome, and the incidence of cardiovascular death is increased in patients with liver transplantation with PTDM ( 3 ). Post-transplant DM shares some pathophysiological characteristics with type 2 diabetes, such as impaired peripheral glucose uptake and insulin secretion, increased hepatic glucose production, and impaired incretin secretion. However, transplant patients have independent risk factors that decrease glucose tolerance, causing PTDM ( 4 ). The use of immunosuppressants also contributes to increasing the risk of PTDM. This is especially true for calcineurin inhibitors such as tacrolimus and cyclosporine. They reduce the secretory capacity of β-cells and increase insulin resistance ( 5 , 6 ). Other drugs commonly associated with PTDM are corticosteroids, particularly in higher doses, usually prescribed in the initial months after transplantation ( 5 ). The surgical stress also has a deleterious effect on pancreatic β-cells, causing a metabolic disorder that releases catabolic hormones and reduces insulin secretion, resulting in hyperglycemia ( 6 , 7 ).

Several studies have shown that the PTDM onset occurs between 6 months to 1 year after transplant ( 8 , 9 ). Implementing strategies to avoid the development of modifiable risk factors in those patients, maintaining glycemic control in the target range, is essential, especially in the first year after the operation. Data are scarce in the literature related to the identification of risk factors associated with diabetes after liver transplantation.

This study aims to identify risk factors associated with diabetes after liver transplant. We hypothesize that in the setting of liver transplantation, risk factors are different from other solid organ transplantations. Specifically, some pre-transplant conditions could increase the risk for PTDM, such as liver steatosis, inflammation, and hepatitis C infection. Additionally, the greater use of calcineurin inhibitors in liver transplantation compared to other solid organ transplants could also be a significant risk factor.

## MATERIALS AND METHODS

### Study design

This cross-sectional study was conducted at the Gastrocentro Post-Liver Transplant Clinic at the University of Campinas, a service registered within the Brazilian National Transplant System. The service has a good reputation as one of the highest volume centers in liver transplantation in Brazil and it has performed more than 1,000 procedures to date. The surgical team comprises two experienced surgeons with a high-volume surgical record, an assistant surgeon, and one or two medical residents.

Only deceased-donor procedures are performed. Briefly, the operation consists of removing and preparing the donor's liver after a case of encephalic death is declared. The donor's liver is inspected to ensure minimal conditions for the transplantation, and the medical records are checked for potential exclusion criteria. Then, the liver is transported in appropriate preserving conditions as fast as possible to the surgical wing at the university's hospital, where the receiver is already waiting. The organ is then implanted, and vascular and biliary anastomoses are performed. From removing the cirrhotic liver to the implant in the receiver, the entire procedure takes from 8 to 12 hours.

This research covered the population of patients from 1990 onwards who were followed at the Gastrocentro Post-Transplant Liver Outpatient Clinic and had appointments from September to November 2019. This study was approved by the research ethics committee, CAAE: 22575319.4.0000.5404.

**Figure 1:**
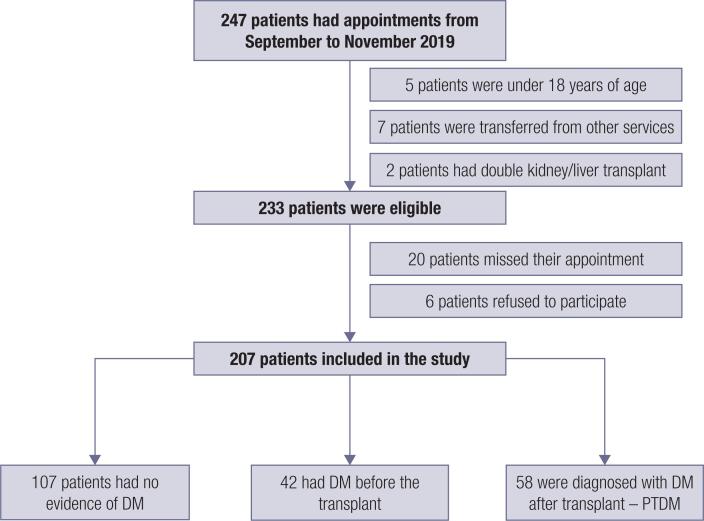
Study flowchart.

All patients older than 18 who underwent orthotopic liver transplant were included in our study. Patients who transferred from other services or who were younger than 18 were excluded. The research sample was selected based on a convenience sample of medical records of patients scheduled for their routine appointments from September to November 2019, and patients were divided into three groups: patients without DM who underwent liver transplant and remained euglycemic at the time of analysis, patients with pre-transplant DM, and patients diagnosed with DM after the transplant. Patients who transferred from other services or those who had another transplant (e.g., kidney and liver transplant) were also excluded. Only patients who attended their appointments were included ( Figure 1 ).

Data collection was performed by chart review, and the analyzed data were sex, age, diagnosis of diabetes pre- or post-transplant, transplant date, time since transplant, reason for transplant, time since diabetes diagnosis, time after transplant for diagnosis of diabetes, cirrhosis due to alcohol, smoking status, current weight, height, body mass index, current diabetes medications, initial HbA1c, last HbA1c, hypertension, hypercholesterolemia, hypertriglyceridemia, laboratory data (fast plasma glucose, creatinine, estimated glomerular filtration rate [GFR-CKD-EPI], total cholesterol, HDL, LDL, triglycerides, and TSH), current immunosuppressive drugs and dose (mycophenolate, prednisolone, tacrolimus, everolimus, cyclosporine, azathioprine, and sirolimus) and the maximum dose already used, and hepatitis B and hepatitis C diagnosis and treatment before or after the transplant. Variables related to hypercholesterolemia and hypertriglyceridemia were analyzed in a dichotomous and continuous manner. Alcohol intake was not quantified and was considered positive if the cause for liver transplant included alcohol use. Hypertension was confirmed if a patient presented abnormal blood pressure during the consultation or was on antihypertensive medications. Hypercholesterolemia was defined if total cholesterol was greater than 200 mg/dL, LDL cholesterol was greater than 130 mg/dL in nondiabetic patients or greater than 100 mg/dL in patients with DM, or when the patient was on statin or ezetimibe. Hypertriglyceridemia was confirmed if triglycerides were greater than 150 mg/dL or the patient was on any fibrate. Smoking was considered positive in any patient who was a current or previous smoker. Immunosuppressant use was considered when the drug was used during at least the interval between two appointments (usually 3 months for each appointment, total of at least 6 months of exposure).

Patients who met the following criteria at least 6 months after transplantation were diagnosed with PTDM: two separate values of fasting glycemia greater than 126 mg/dL or 2-hour glucose levels greater than 200 mg/dL after a 75 g oral glucose challenging test. After 1 year of transplantation, HbA1c test greater than 6.5% was also considered for the diagnosis of PTDM. If hyperglycemia was detected after the surgical procedure but remitted within 6 months and did not recur, then these patients were not diagnosed as having PTDM.

Biopsy data from implant and explant were also routinely collected at the time of transplantation and/or in the organ rejection evaluation. We also analyzed the presence of cirrhosis, HCC (tumor size, differentiation, and invasion), interface activity, preservation before and after transplantation of the implant, etiology, hepatic steatosis in the recipient, and presence of rejection. Interface activity refers to the microscopic finding of lymphocytes and other inflammatory cells leaving the portal tracts and surrounding hepatocytes at the interface of the parenchyma and connective tissues of the portal zone, formerly known as “piecemeal necrosis.”

### Statistical analysis

The data were presented as measures of position and dispersion (median and interquartile range) when numerical or as frequency and percentage when categorical. The comparison between the three groups of patients was performed using the Kruskal-Wallis test with the Bonferroni post hoc test. Categorical data were compared using the χ^2^ test or Fisher's exact test when the frequency of a given value was less than 5. The influence of clinical or laboratory factors on the development of PTDM and its complications was performed through multivariate logistic regression analysis. Two models were evaluated: the first comparing patients without DM versus all patients with DM (pre- and post-transplant) and the second comparing patients without DM exclusively with patients with PTDM alone. The software used for data analysis was SPSS for Mac, version 20.0. A value of *P* < 0.05 was considered statistically significant.

## RESULTS

### Baseline characteristics

Two hundred and forty-seven patients were screened, and the final sample consisted of 207 patients, with 107 subjects without diabetes, 42 with pre-transplant diabetes, and 58 with PTDM ( Figure 1 ). Most patients were male, with 72.9% in patients without DM, 64.3% in patients with pre-transplant DM, and 75.9% in patients with PTDM. The leading cause for liver transplant was hepatitis C in all groups, followed by cirrhosis secondary to alcohol intake and other causes. The median age in patients without DM, pre-transplant DM, and PTDM was 56 (47-63), 62 (56.75-66.50), and 60 (54-63.25), respectively. A significant statistical difference in age was seen between patients without DM and the PTDM group ( *P* = 0.001). Average transplant time was longer in patients with PTDM compared to patients with pre-transplant DM: 6.5 years (range = 2.46-11.04) versus 2.5 years (range = 1.08-4.38; *P* = 0.001). Prevalence of hypertension and hypercholesterolemia showed a statistically significant difference between groups, being higher in patients with pre-transplant diabetes, followed by patients with PTDM. Hypertriglyceridemia was more common among patients with pre-transplant DM than it was in other groups. Hepatitis C and cirrhosis due to alcohol were more frequent in patients with PTDM. When analyzing laboratory tests, triglyceride levels were higher in patients with pre-transplant DM than they were in patients without DM ( *P* < 0.001). Serum creatinine in patients with pre-transplant DM was higher compared to the PTDM group ( *P* = 0.026) and patients without DM ( *P* = 0.019). The estimated GFR also showed lower values in patients with pre-transplant DM than were seen in other groups ( *P* = 0.015 vs. the PTDM group and *P* = 0.002 vs. patients without DM). Table 1 shows the detailed clinical and laboratory characteristics of all groups.

**Table 1:** Clinical characteristics and laboratory data of liver transplantation patients without diabetes, with pre-transplant diabetes, and with post-transplant diabetes

Variables	Patients without DM n = 107	Pre-transplant DM n = 42	PTDM n = 58	p
Gender (male)	78 (72.9%)	27 (64.3%)	44 (75.9%)	0.425
Age (years)	56 (47-63)	62 (56.75-66.50)	60 (54-63.25)	0.001 a
Transplant duration (years)	4 (2-8)	2.5 (1.08-4.38)	6.5 (2.46-11.04)	0.001 c
Diabetes duration (years)	-	10 (8-19)	2.5 (1-4.25)	<0.001
Weight (kg)	75 (63.5-86.90)	71 (65-84.50)	74 (67.75-86)	0.670
Body mass index (kg/m2)	26.15 (23.15-28.09)	27.44 (24.54-30.46)	26.53 (24.46-28.72)	0.309
Cirrhosis due to alcohol	23 (21.5%)	12 (28.6%)	23 (41.8%)	0.025
Hepatocellular carcinoma secondary to alcohol intake	14 (13.1%)	8 (19%)	12 (20.7%)	0.397
Smoking	4 (7.7%)	4 (13.8%)	6 (19.4%)	0.290
Hypertension	38 (35.5%)	26 (61.9%)	30 (51.7%)	0.008
Hypertriglyceridemia	28 (26.2%)	24 (57.1%)	15 (25.9%)	0.001
Hypercholesterolemia	25 (23.4%)	22 (52.4%)	16 (27.6%)	0.002
Hepatitis B	14 (13.3%)	2 (4.9%)	3 (5.2%)	0.126
Hepatitis C	38 (35.8%)	15 (37.5%)	35 (60.3%)	0.007
HbA1c (%)	5.3 (4.7-5.45)	6.9 (6-7.75)	6.5 (5.6-7)	0.002 a , b
Glycemia (mg/dL)	93 (84-105)	127 (84.5-151)	117 (98-158)	<0.001 a , b
Creatinine (mg/dL)	1.1 (0.88-1.30)	1.25 (1.06-1.66)	1.08 (0.89-130)	0.013 a , c
GFR (mL/min)	78.72 (55.12-104.99)	60.43 (43.50-73.71)	75.63 (57.40-96.59)	0.002 a , c
Total cholesterol (mg/dL)	153 (134-179)	170 (126.5-209.50)	161 (130-183.5)	0.310
HDL (mg/dL)	42 (34-52.5)	39 (35-50)	44 (35.25-50)	0.558
LDL (mg/dL)	90.5 (67.25-108.75)	93.5 (65 – 118.9)	91.50 (66.75-110.5)	0.974
Triglycerides (mg/dL)	101 (75.5-150)	160 (106.5-256)	114 (88.5-169.5)	<0.001 a
TSH (μUI/mL)	2.37 (1.65-3.9)	2.54 (1.84-4.7)	2.74 (1.69-4.11)	0.779

Values are presented as median (interquartile range) for continuous variables and frequency (percentages) for categorical variables. DM: diabetes mellitus; PTDM: post-transplant diabetes mellitus.

a Post hoc test difference between the group of patients without DM and pre-transplant DM.

b Post hoc test difference between the group of patients without DM and PTDM.

c Post hoc test difference between the group of patients with pre-transplant DM and PTDM.

Regarding exposure to immunosuppressant drugs, there was a higher frequency of tacrolimus prescription in patients without DM compared to patients with pre-transplant DM and patients with PTDM (86.9% vs. 68.3% and 86.9% vs. 84.5%; *P* = 0.02). There was a higher frequency of exposure to ciclosporin in patients with pre-transplant DM compared to the other groups ( *P* = 0.005). Table 2 shows detailed information about exposure and doses of immunosuppressants in all groups.

**Table 2:** Exposure to immunosuppressants and dose of those medications in liver transplantation patients without diabetes, with pre-transplant diabetes, and with post-transplant diabetes

Variables	Patients without DM	Pre-transplant DM	PTDM	p
Mycophenolate	81 (75.7%)	33 (80.5%)	36 (62.1%)	0.080
Dose of mycophenolate (mg)	720 (720-720)	720 (720-720)	720 (720-720)	0.401
Prednisone	27 (25.2%)	15 (36.6%)	22 (39.3%)	0.134
Tacrolimus	93 (86.9%)	28 (68.3%)	49 (84.5%)	0.025
Dose of tacrolimus (mg)	4 (2-6)	5 (2-7)	2 (2-4)	<0.001 a , b
Maximum dose of tacrolimus (mg)	8 (6-10.5)	8 (6-10)	7 (5-8)	0.038 b
Everolimus	20 (18.7%)	13 (31.7%)	12 (20.7%)	0.223
Dose of everolimus (mg)	1 (1-2)	1.5 (0.69-1.81)	0.88 (0.5-1.5)	0.226
Cyclosporine	6 (5.6%)	10 (24.4%)	9 (15.5%)	0.005
Dose of cyclosporine (mg)	150 (75-175)	150 (125-200)	100 (87.50-150)	0.497
Maximum dose of cyclosporine (mg)	150 (150-225)	262.5 (187.5-325)	175 (118.75-325)	0.368
Azathioprine	9 (8.4%)	5 (12.2%)	6 (10.5%)	0.765
Dose of azathioprine (mg)	50 (50-100)	50 (50-75)	100 (50-100)	0.451
Sirolimus	2 (1.9%)	2 (4.9%)	3 (5.3%)	0.442
Dose of sirolimus * (mg)	1.5 (1.0)	1.0 (1.0)	0.75 (0.5)	0.368

Values are presented as median and interquartile range for continuous variables and percentages for categorical variables.

* Less than three patients were on sirolimus in each group.

a Post hoc test difference between the group of patients without DM and pre-transplant DM.

b Post hoc test difference between the group of patients without DM and PTDM.

### Anatomopathological data

A significant difference in the evaluation of interface activity was seen, being more severe in patients without DM and in the PTDM group than it was in patients with pre-transplant DM: 60.8% versus 67.7% versus 37.5%, respectively ( *P* = 0.032). There were no statistically significant differences between patients without DM, pre-transplant DM, and PTDM, respectively, in HCC size (2.6 cm vs. 3 cm vs. 3 cm; *P* = 0.409) or degree of tumor differentiation ( *P* = 0.167). Liver tissue preservation indexes before and after the transplant (before transplant: 23.9% vs. 20.8% vs. 33.3%, *P* = 0.708; after transplant: 61.2% vs. 58.1% vs. 53.8%, *P* = 0.807) were also not different between the three groups.

There was a higher prevalence of steatosis in the recipients with pre-transplant DM than was found in patients without DM and with PTDM (11.6% vs. 59.1% vs. 8.3%; *P* < 0.001). However, no difference was seen regarding HCC invasion (21.4% vs. 30% vs. 13%; *P* = 0.397) and rejection (10.2% vs. 22.7% vs. 25.8%; *P* = 0.073).

Logistic regression was performed in two different models. The first was used to analyze patients without diabetes versus patients with pre-transplant diabetes in conjunction with those with PTDM. The second was performed among patients without diabetes and PTDM, excluding those with pre-transplant diabetes. In the first model, we were able to identify the following independent risk factors for DM: cirrhosis due to alcohol, hepatitis C, and triglycerides. In the second multivariate analysis model, the independent risk factors for PTDM were cirrhosis due to alcohol, hepatitis C, and prednisone exposure. Table 3 shows the details of the two models of multivariate analysis.

**Table 3:** Multivariate analysis assessing independent risk factors for DM (model 1) and PTDM (model 2)

Model 1 – All patients included (no DM versus all DM)			
Risk factors	Odds Ratio	95% CI	p
Cirrhosis due to alcohol	2.90	1.47-5.7	0.002
Hepatitis C	2.02	1.08-3.75	0.027
Triglycerides	1.00	1.0-1.01	<0.001

Model 2 – patients without DM versus PTDM			
Risk factors	Odds Ratio	95% CI	p
Cirrhosis due to alcohol	3.77	1.69-8.4	0.001
Hepatitis C	3.35	1.57-7.09	0.002
Prednisone	2.77	1.26-6.08	0.011

DM: diabetes mellitus; PTDM; post-transplant diabetes mellitus.

## DISCUSSION

In our study, alcoholic cirrhosis was an independent risk factor for PTDM. Other authors have shown similar results, such as Skladaný and cols. ( 10 ), who performed a retrospective study in 2019 with 102 patients evidencing alcoholic liver disease as a new risk factor for PTDM. Liu and cols. ( 11 ) found alcoholic hepatitis as a predictive factor among 189 patients with PTDM. However, in a meta-analysis carried out by Li and cols. ( 12 ) in 2015, no association was found between HCC due to alcohol use and PTDM.

In our study, hepatitis C was also an independent risk factor for PTDM. Chronic hepatitis C infection is known to be associated with impaired glucose metabolism. The effect occurs in a two-way fashion, because infection by the virus is more common in patients with diabetes, and patients infected by hepatitis C virus have a higher prevalence of diabetes. Hepatitis C virus is linked to insulin resistance, contributing to the morbidity and mortality associated with hepatitis C infection. The virus is responsible for peripheral and hepatic insulin resistance due to increased TNF-α secretion and other pro-inflammatory cytokines that interfere in post-receptor insulin signaling pathways ( 13 ). Several studies have confirmed this relationship and pointed out the disease as an independent risk factor for diabetes and PTDM. A meta-analysis performed by White and cols. ( 14 ) with retrospective and prospective studies concluded that hepatitis C infection is related to an increased risk of PTDM both in comparison with uninfected patients and with other liver infections, such as hepatitis B ( 15 ).

In our multivariate analysis between patients with diabetes versus patients without diabetes, triglyceride level was an independent risk factor associated with DM. It is well established that in non-transplant patients, high levels of triglycerides are associated with an increased risk of type 2 diabetes, because ectopic lipid deposition in the liver, pancreatic islets, and skeletal muscle is responsible for β-cell dysfunction and worsening insulin resistance. A study with liver transplant patients showed a significant association between preoperative triglyceride levels and a higher incidence of PTDM, although only in men; an increment of 1 mmol/L in triglyceride levels increased the risk of PTDM by 37% ( 16 ).

In our logistic regression between patients without DM and PTDM, exposure to prednisone was another independent risk factor for PTDM. Corticosteroids have a cytotoxic and antiproliferative effect on β-cell and interfere with insulin signaling pathways, thus reducing glycogen synthesis, GLUT4 translocation, and glucose uptake by skeletal muscle. This results in hyperglycemia and reduced insulin secretion ( 17 ). Several studies have proven the diabetogenic effect and the higher incidence of PTDM in immunosuppression schemes with corticosteroids, especially if prolonged and higher doses are used. Castedal and cols. ( 18 ) performed an immunosuppression protocol free of corticosteroids and with low doses of tacrolimus after liver transplantation, resulting in a lower incidence of PTDM. A study carried out from the Organ Procurement and Transplantation Network/United Network for Organ Sharing transplant database with 20,172 liver transplant recipients showed immunosuppression with corticosteroids as a risk factor for PTDM ( 19 ). In our comparative analysis, exposure to tacrolimus was less common in PTDM, probably due to our service's prescription bias in preserving those patients with impaired glucose metabolism from a known diabetogenic medication, in which case they were prescribed cyclosporine. Robust evidence in the literature shows CNIs are diabetogenic. Mechanisms include apoptosis of β-cells by reducing the transcription of survival factors and reduced expression of GLUT2 and glucokinase, generating a deficit in insulin secretion. Additionally, there is an induction of peripheral insulin resistance by exchanging type 1 muscle fibers with type 2, which are less sensitive to insulin ( 17 ). Tacrolimus is the CNI most often related to PTDM and it has been identified as a predictive risk factor in meta-analyses ( 12 , 20 ). Exposure to higher doses of tacrolimus progressively increases the risk of PTDM; Song and cols. ( 6 ) found a serum level of tacrolimus of 5.89 ng/mL, 6 months after transplant, was a cutoff for higher risk of PTDM. Regarding cyclosporine, the data are controversial. Sánchez-Pérez and cols. ( 21 ) reported that patients treated with tacrolimus were four times more likely to develop PTDM than those treated with cyclosporine were. On the other hand, Xue and cols. ( 22 ) found that cyclosporine was a risk factor for PTDM, but only in univariate analysis and in a study comparing liver receptors with and without liver steatosis.

There was a significant difference between the groups regarding the presence of hypertension and hypercholesterolemia, being higher in patients with pre-transplant diabetes, followed by patients with PTDM. This may suggest the development of diabetes, hypertension, and hypercholesterolemia are related to the same risk factors, such as obesity and metabolic syndrome, a fact that may explain the finding in our study. Regarding hypertension, some studies have found a similar relationship. Terto and cols. ( 23 ) have shown pre-transplant hypertension as a risk factor for PTDM. Algarem and cols. ( 24 ) showed that DM and hypertension are common findings after solid organ transplants. It should be noted that in liver transplant recipients, a relationship exists between cholesterol levels and the use of corticosteroids due to increased activity of acetyl-CoA carboxylase and fatty acid synthesis. In addition, due to CNI exposure, decreased excretion of biliary cholesterol and blockade of LDL cholesterol receptors are frequent and more common with ciclosporin than they are with tacrolimus ( 25 ).

Regarding creatinine, we found higher values in patients with pre-transplant diabetes, which can be explained by the longer duration of DM, leading to progressive loss of renal function if adequate metabolic control is not achieved. Additionally, creatinine values may have worsened due to the surgical insult of the extensive procedure and the use of nephrotoxic medications. Comparable results were obtained when using estimated GFR. Trail and cols. ( 26 ) showed similar results with declining renal function with elevated creatinine and urea in patients with pre-transplant DM.

BMI was not a risk factor in our research, as seen in other studies with liver and kidney transplant recipients. This could be explained by the fact that in patients with liver failure, BMI calculation can be confused by ascites and malnutrition linked to the baseline disease. Leonard and cols. ( 27 ) reported that 11% to 20% of patients were classified as having a lower BMI when correcting for ascites volume. Therefore, the effect of BMI on PTDM can be underestimated.

In our study, the presence of hepatic steatosis in the recipient was more frequent in patients with pre-transplant DM, data that are compatible with obesity and metabolic syndrome being more frequent in this group. Liver steatosis causes the accumulation of lipids in hepatocytes and impairs insulin signaling, and it worsens insulin resistance by stimulating gluconeogenesis. Interface activity was more severe in patients without DM and in those with PTDM than it was in patients with pre-transplant DM. There is little research about this type of anatomopathological data in the context of liver transplant. Feng and cols. ( 28 ) carried out a study in pediatric liver transplant patients and related the interface activity to subclinical rejection due to T cells’ higher inflammatory activity because it suggests worst outcomes in PTDM, mainly graft rejection. It must be emphasized that most of our sample had hepatitis C as the reason for transplant, which is known to be related to different degrees of interface activity and chronic inflammatory process. These inflammatory changes alter hepatic glucose metabolism and insulin resistance, predisposing patients to the development of PTDM.

It is essential to highlight the impossibility of ascertaining causation in the risk factors associated with PTDM found in our study due to the cross-sectional nature. Nonetheless, the associations found make pathophysiological sense, and they deserve to be better investigated in further prospective studies. In the meantime, it would be prudent to have a higher degree of awareness of PTDM in patients with these characteristics during post-transplant follow-up. Another important limitation of our study is the selection of patients by convenience sampling, which could introduce selection bias. We tried to minimize this risk by analyzing the records in a span of 3 months in which most patients currently under care would have appointments as a way to screen the most patients followed-up in the service.

In conclusion, our study presented the following independent risk factors for PTDM in liver recipients: hepatitis C, cirrhosis due to alcohol, and prednisone use. Other factors associated with a higher frequency of DM were age, time since transplantation, hypertriglyceridemia, interface activity, steatosis in the recipient, creatinine, hypertension, hypertriglyceridemia, and hypercholesterolemia. Although causation cannot be ascertained in a transversal study, the associations found between the aforementioned factors and PTDM should prompt special attention to the risk of PTDM development. Approaches such as nutritional advice, weight management, and the use of more frequent and sensitive diagnostic methods for DM (e.g., the 75 g glucose challenge test) could be considered in patients after liver transplant when there is a background of hepatitis C infection, cirrhosis due to alcohol abuse, and/or exposure to prednisone after the procedure. Our findings should be explored and confirmed in prospective studies involving liver recipients and underline the specificity and differences of risk factors for PTDM related to each organ transplant.
